# Ultra‐Narrow Phosphorene Nanoribbons Produced by Facile Electrochemical Process

**DOI:** 10.1002/advs.202203148

**Published:** 2022-09-06

**Authors:** Usman O. Abu, Sharmin Akter, Bimal Nepal, Kathryn A. Pitton, Beth S. Guiton, Douglas R. Strachan, Gamini Sumanasekera, Hui Wang, Jacek B. Jasinski

**Affiliations:** ^1^ Conn Center for Renewable Energy Research University of Louisville Louisville KY 40292 USA; ^2^ Department of Mechanical Engineering University of Louisville Louisville KY 40292 USA; ^3^ Department of Physics and Astronomy University of Louisville Louisville KY 40292 USA; ^4^ Department of Chemistry University of Kentucky 125 Chemistry–Physics Building Lexington KY 40506‐0055 USA; ^5^ Department of Physics and Astronomy University of Kentucky 177 Chemistry–Physics Building Lexington KY 40506‐0055 USA

**Keywords:** field‐effect transistors, Na intercalation, nanoribbons, n‐type, phosphorene

## Abstract

Phosphorene nanoribbons (PNRs) have inspired strong research interests to explore their exciting properties that are associated with the unique two‐dimensional (2D) structure of phosphorene as well as the additional quantum confinement of the nanoribbon morphology, providing new materials strategy for electronic and optoelectronic applications. Despite several important properties of PNRs, the production of these structures with narrow widths is still a great challenge. Here, a facile and straightforward approach to synthesize PNRs via an electrochemical process that utilize the anisotropic Na^+^ diffusion barrier in black phosphorus (BP) along the [001] zigzag direction against the [100] armchair direction, is reported. The produced PNRs display widths of good uniformity (10.3 ± 3.8 nm) observed by high‐resolution transmission electron microscopy, and the suppressed *B*_2g_ vibrational mode from Raman spectroscopy results. More interestingly, when used in field‐effect transistors, synthesized bundles exhibit the n‐type behavior, which is dramatically different from bulk BP flakes which are p‐type. This work provides insights into a new synthesis approach of PNRs with confined widths, paving the way toward the development of phosphorene and other highly anisotropic nanoribbon materials for high‐quality electronic applications.

## Graphical abstract

Black phosphorous (BP) flakes are nanostructured via electrochemical intercalation of Na^+^ ions into bundles of phosphorene nanoribbons (PNRs). The large diffusion barrier of Na^+^ ions along the armchair direction leads to a well‐defined columnar intercalation of Na^+^ ions in BP, resulting in zigzag‐oriented columns of disordered material and PNRs in‐between. The sonication is then used to separate the PNRs.

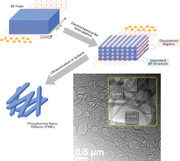

## 1 Introduction

The impressive physics exhibited by graphene and its derivatives after its successful isolation in 2004 has sparked the strong interests of researchers in the development of novel 2D layered materials and the subsequent exfoliation of their layers.^[^ ^1^ ^]^ While 2D materials confine charge carriers to a plane (electron motion is not confined in two dimensions with only one dimension quantized), 1D structures, such as nanowires and nanoribbons (NRs),^[^ ^2^ ^]^ localize carriers in one more dimension^[^ ^2^ ^]^ (electron motion is not confined in one dimension with two dimensions quantized) which leads to additional unique properties, including higher mobility, strain tunable characteristics, high optical absorption, high density of states, enhanced exciton binding energy, and improved surface scattering for electrons.^[^ ^3^ ^]^ NRs have also high concentration of edge sites, which are often responsible for excellent catalytic performance and allow an easy functionalization and further tuning of properties toward high performance applications^[^ ^2^ ^]^ in fields such as energy storage, hydrogen generation, and sensors.^[^ ^4^ ^]^ The nanostructuring of 2D layered materials into 1D nanoribbons, not only results in significant redesigning of material's density of states and band structure but also creates a high‐density of edge sites. These advanced features lead to interesting properties such as high catalytic activity and ease for functionalization; thus, opening up lots of novel possibilities in a throng of applications.^[^ ^2^ ^]^

Among a plethora of post‐graphene 2D layered materials, phosphorene which is exfoliated from black phosphorous (BP) has been a subject of intense research since its maiden isolation in 2014.^[^ ^5^ ^]^ This is due to phosphorene's unique properties, including its high charge carrier mobility (2000 cm^2^ V^−1^ s^−1^), thickness‐dependent bandgap (0.3–2.0 eV), and high in‐plane anisotropy.^[^ ^1^, ^6^ ^]^ Phosphorene degrades in hours by the combined action of moisture and oxygen upon exposure to ambient conditions because of reactive lone pair of electrons in P atoms.^[^ ^7^ ^]^ This has been a drawback to its device compliance in lots of applications. Strategies like encapsulation,^[^ ^8^ ^]^ surface passivation,^[^ ^9^ ^]^ surface functionalization,^[^ ^10^ ^]^ and doping^[^ ^7^, ^11^ ^]^ have been developed with varying degrees of success to help enhance the stability of phosphorene. Phosphorene starts degrading from the surface^[^ ^12^ ^]^ as oxygen molecules approach with an exothermic energy of −4.07 eV per molecule.^[^ ^12^ ^]^ Cutting phosphorene sheets into nanoribbons leaves highly active and unstable edges;^[^ ^13^ ^]^ thus, potentially leaving them chemically less stable than phosphorene sheets. Passivating phosphorene nanoribbons (PNRs) edges with functional groups like hydrogen could potentially improve its stability as observed in ab initio calculations.^[^ ^13^, ^14^ ^]^ PNRs display even more impressive properties (e.g., increase bandgap, modified electronic structure, and higher density of states) due to quantum confinement effects, and high density of edge sites. PNRs have also been predicted to exude excellent properties like polarization‐dependent anisotropic response, exceptional mechanical properties, and highly active bonding sites.^[^ ^2^, ^15^ ^]^ Quantum confinement and diminished dielectric screening causes excitons in atomically thin semiconductors like PNRs to exhibit binding energies an order of magnitude larger than their bulk counterpart.^[^ ^16^ ^]^ Consequently, they show high promise for applications in electronic devices, optics, magnetism, and catalysis.^[^ ^2^, ^17^ ^]^ Given their high exciton binding energies, tunable band gaps, solution‐based processing, and very high carrier mobilities, PNRs are highly promising materials for optoelectronics. Recently, MacDonald et al.^[^ ^4^ ^]^ have demonstrated the potential of PNRs for photovoltaic applications. They have incorporated PNRs into perovskite solar cells and demonstrated improved efficiency of these devices due to the enhanced electrical transport between the light‐absorbing perovskite layer and a semiconducting polymer. PNRs are also predicted to host several exotic states^[^ ^18^ ^]^ and may play an important role in several fundamental areas of condensed matter physics. In addition to topologically‐protected edge states,^[^ ^19^ ^]^ some of the other exotic properties predicted for these structures include spin‐density waves,^[^ ^20^ ^]^ strain‐dependent antiferromagnetism,^[^ ^21^ ^]^ and half‐metallic behavior^[^ ^22^ ^]^ which could be relevant to spintronics applications,^[^ ^23^ ^]^ the spin‐dependent Seebeck effect,^[^ ^24^ ^]^ that could help to advance thermoelectric technologies, and a large singlet–triplet splitting,^[^ ^18^ ^]^ that could potentially be relevant to quantum information. The crossed Andreev reflection^[^ ^25^ ^]^ has also been recently investigated theoretically in structures based on such nanoribbons.

Starting in 2016, initial attempts of producing PNRs, such as etching,^[^ ^26^ ^]^ electro‐beam sculpting,^[^ ^26^ ^]^ and electro‐beam lithography^[^ ^26^ ^]^ have been explored. However, these expensive and complicated approaches yielded nanoribbons with limited lengths and often stacked together. Only recently, more efficient and cost‐effective top‐down exfoliation approaches have been attempted toward the synthesis of PNRs. In 2019, Watts et al.^[^ ^18^ ^]^ first have produced high‐quality PNRs by intercalating bulk BP with Li ions via a low‐temperature, ammonia‐based method, and then mechanically exfoliating Li‐intercalated BP into nanoribbons in stable liquid dispersions. Although this method has been used by MacDonalds et al.^[^ ^4^ ^]^ to synthesize PNRs nanoribbons applied in photovoltaic devices, the cryogenic (−50 °C) processing requirements of this process make it costly and present a challenge for scalability.^[^ ^4^ ^]^ Subsequently, Liu et al.^[^ ^27^ ^]^ have synthesized phosphorene nanobelts (PNBs) electrochemically in an oxygen assisted intercalation process. While the thickness of most of the belts produced was less than 3 nm, the impact of oxygen molecules at the edges of as‐prepared PNBs is problematic. Yu et al.^[^ ^15^ ^]^ have used a dual electrochemical set‐up based on quaternary ammonium electrolyte to produce PNRs that had relatively higher aspect ratio (≈100) than those reported by Watts and Liu. However, majority of the widths of the ribbons produced were still in the micron scale. Very recently, Macewicz et al.^[^ ^28^ ^]^ have complimented a chemical vapor transport (CVT) with mechanical exfoliation to produce larger BP nanoribbons and nanobelts that had a length–width ratio in a few hundred range (with width of 1.5 µm and length of 500 µm). Such lager dimensions may limit the active sites available for chemical bonding and modification.^[^ ^2^ ^]^

Recently, we have reported that the electrochemical Li intercalation in BP was highly anisotropic, and the Li^+^ diffusion along the channels of the puckered structure of BP lattice led to the grove formation and segmentation of BP flakes into weakly connected nanoribbon‐like strips along the zigzag direction.^[^ ^29^ ^]^ In addition, Kim et al. have observed the anisotropic diffusional behaviors for both Li^+^ and Na^+^ that favor the diffusion along the zigzag direction over the armchair direction in BP; the diffusion barrier of Na^+^ along the armchair direction (268 meV) is significantly larger than that of Li^+^ (156 meV).^[^ ^30^ ^]^ On the basis of these important works, we generate the hypothesis that the anisotropic Na^+^ diffusion barrier in black phosphorus (BP) along [001] zigzag direction against [100] armchair direction possibly provides the great chance to produce PNRs and the narrow stripes along zigzag direction induced by anisotropic Na intercalation is possibly the precursors to produce PNRs, which show unique performance.

Herein, we demonstrate a low‐cost and feasibly scalable two‐step electrochemistry‐based method for synthesizing PNRs with confined widths (10.3 ± 3.8 nm) and relatively uniform lengths (250 ± 156 nm), dramatically narrower than PNRs in most of the previous methods. In this two‐step approach, BP flakes are first nanostructured through an electrochemical discharge process into bundles of parallel PNRs separated from each other by regions of highly disordered phosphorous, then followed by an ultrasonic treatment in dimethylformamide (DMF) solvent to separate the PNR bundles into individual and well‐isolated PNRs. The produced PNRs show a significantly confined structure with the suppressed *B*_2g_ vibrational mode, as revealed by Raman spectroscopy. Furthermore, unlike in the case of phosphorene or BP devices, a field‐effect transistor (FET) prepared from a bundle of our unseparated PNRs exhibits the typical n‐type behavior.

## 2 Results

**Figure 1:**
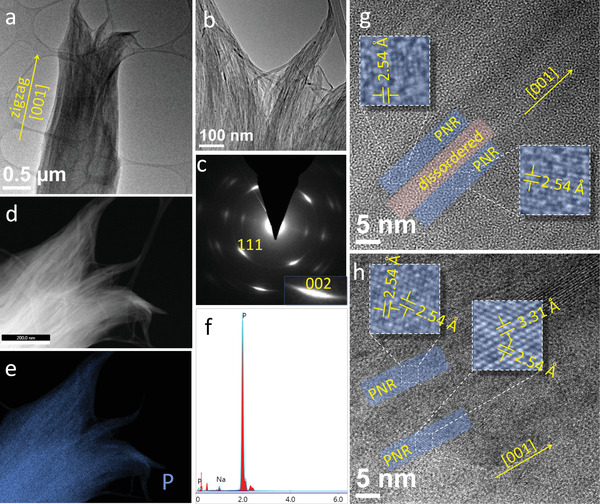
Structural characterization of an unseparated PNR bundle obtained from a BP flake through electrochemical Na intercalation: a) Low‐ and b) high‐magnification TEM image of a typical PNR bundle. c) SAED of the same PNR bundle. Insets show the (002) diffraction spot having a clear arc form. d) STEM image, e) EDS phosphorous map, and f) EDS spectrum of the same PNR bundle. g,h) HRTEM images of such a PNR bundle. Sections of example PNRs are shown with blue strips, while the disordered region between two PNRs is shown using a pink strip.

To obtain a better understanding of our PNR fabrication method and to characterize the PNRs produced by this method, a detailed structural and elemental analysis was conducted before and after the ultrasonication step, that is, after the PNRs were separated from each other. For these measurements, the obtained samples were dispersed in DMF, drop‐casted on holey carbon‐on‐copper grids, and analyzed using transmission electron microscopy (TEM). A representative set of the data from the obtained BP sample after electrochemical intercalation is shown in **Figure** **1**. Figure 1a,b shows a low‐ and high‐magnification TEM image of a typical unseparated bundle of PNRs, respectively. The brush‐like morphology of the bundle is the result of nearly parallel PNRs separated one from each other by narrow columnar regions of highly disordered phase. Selected‐area electron diffraction (SAED) was used to analyze the crystallinity of the PNRs located between these amorphous regions. The SAED pattern of the bundle shown in Figure 1a,b is presented in Figure 1c. The strongest diffraction reflections visible in this pattern are the (002)‐ and (111)‐type reflections of BP. However, instead of sharp diffraction spots, these reflections are seen as arcs due to slight deviations of PNRs from being exactly parallel to each other. Nevertheless, from the comparison between TEM images and the corresponding SAED patterns it was determined that PNRs are parallel to their [001] (i.e., zigzag) crystallographic direction. The majority of Na‐intercalated BP flakes observed in TEM had the morphology of unseparated bundles of PNRs, as shown in a few examples included in Figure S1, Supporting Information. The edges of the PNRs are terminated with an amorphous layer (Figure S2, Supporting Information) which could help to passivate the edges and improve stability. A follow‐up systematic degradation study is however needed to better understand the role of this amorphous layer on nanoribbon stability. The nanoribbons produced by this method are in the few nm thickness range as indicated from high‐resolution TEM (HRTEM) (Figure S3, Supporting Information) and atomic force microscopy (AFM) (Figure S4, Supporting Information).

Furthermore, elemental analysis using energy dispersive X‐ray spectroscopy (EDS) confirmed that PNRs contained mainly phosphorous. This is evident from the elemental mapping (Figure 1e) and EDS spectrum (Figure 1f) of the bundle shown in Figure 1a,b. The high‐angle annular dark filed‐scanning TEM (HAADF‐STEM) image corresponding to the phosphorous map in Figure 1e is shown in Figure 1d. The traces of Na apparent in the EDS spectrum (Figure 1f) are most likely from the highly disordered regions between the PNRs. Since the cut‐off voltage is very low at 0.2 V to achieve full discharge, the reductive products such as Na*_x_* P are expected (at least in the disordered region). However, the weak line of Na in the EDS spectrum suggests that the formed Na*_x_* P compounds are probably dissolved in liquid electrolyte.

The crystalline nature of PNRs was also confirmed using HRTEM. The PNRs showing crystalline lattice fringes were observed separated from each other by regions of disordered phase. Example HRTEM images from the PNR bundle analyzed in Figure 1a–f are shown in Figure 1g,h. The d‐spacing values obtained from the visible lattice fringes were found to be 2.54 and 3.31 Å, which agreed with the d‐spacing values expected for PNRs viewed along the [001] zone axis.

**Figure 2:**
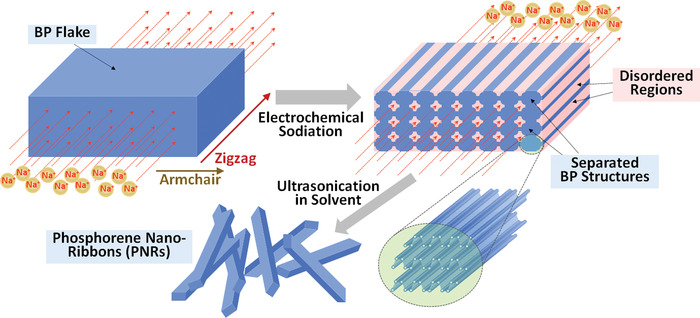
A schematic illustrating a conversation of BP flake (top) into an unseparated PNR bundle (bottom) during electrochemical Na intercalation.

The nanoribbons were produced by anisotropic intercalation of Na^+^ into BP as shown in the model in **Figure** **2**. First, Na^+^ was electrochemically driven into BP layers. Due to a very strong anisotropy of the diffusion coefficient, Na^+^ preferentially moves along the zigzag direction of BP which has longer bond length of 2.244 Å. Other than along the armchair direction where Na^+^ have a high diffusion barrier of 268 meV, Na^+^ diffusion along the zigzag direction has a low energy barrier of 93 meV.^[^ ^30^ ^]^ Such a huge difference is the driving force behind the anisotropic diffusion behavior, resulting in the zigzag‐oriented intercalation channels during sodiation process. On the other hand, the large diffusion barrier of Na^+^ along the armchair direction leads to a well‐defined columnar intercalation of Na^+^ in BP, resulting in the long [001]‐oriented columns of disordered material, as schematically shown in Figure 2. The observed mechanism is similar to the mechanism behind the production of PNRs via Li intercalation driven ionic scissoring described by Watts et al.^[^ ^18^ ^]^ However, the intercalation in the present work is driven electrochemically and not Li^+^ but Na^+^ are intercalated. In the Li^+^ induced process presented by Watts et al.,^[^ ^18^ ^]^ the formation of PNRs is explained by a charge transfer and electron doping which increases over time and eventually causes the bond breaking (or cutting) along the diffusion path. A similar mechanism may take place in our case of electrochemical insertion of Na^+^ ions. Nevertheless, more fundamental studies are needed to understand other, perhaps related phenomena occurring in the material, such as intercalation‐induced strain buildup and its relaxation, as well as defects formation and accumulation. A better understanding of how the observed highly uniform bundles of PNRs are formed is also needed.

Layered materials are typically etched or patterned along a specific direction to form 1D strips as seen in graphene and MoS_2_ nanoribbons.^[^ ^17^ ^]^ The relatively large size of Na^+^ (227 pm) causes the strain and distortion of BP lattice that accumulates during electrochemical intercalation and eventually leads to relaxation and the formation of nanoribbons separated from each other by disordered columnar regions, as shown schematically in Figure 2. This observation is similar to the reported disordered stripes seen by Cheng et al.^[^ ^31^ ^]^ They attributed the amorphization of BP by sodium‐induced reordering of atomic stacks to the breaking of P—P bonds and lattice constraints.

**Figure 3:**
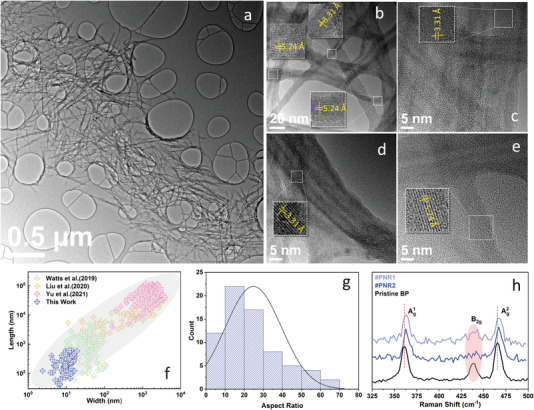
Characterization of PNRs obtained after sonication of Na‐intercalated BP sample: a) Low‐magnification TEM image showing many PNRs on a support holey carbon film. b–e) HRTEM images of individual PNRs. Lattice fringes and their corresponding d‐spacing values are shown in insets. f) Size distribution comparison of PNRs obtained in this work and in previous reports.^[^ ^15^, ^18^, ^27^ ^]^ g) A histogram of the aspect ratio of PNRs shown in (a). h) Raman spectra from two regions of isolated PNRs. A Raman spectrum of BP flake is shown for comparison.

The sonication of the parallel bundle of BP sample after electrochemical intercalation was performed to separate the PNRs, which were then analyzed by HRTEM and Raman spectroscopy. **Figure** **3**a shows the exfoliated PNRs deposited on a holey carbon‐on‐copper grid. The d‐spacing of representative PNRs was measured from HRTEM images shown in Figure 3b–e. Due to the random distribution of PNRs after exfoliation and their occasional twisting, PNRs seen from the top (i.e., along the [010] zone axis) and from the side (i.e., along the [100] direction) can be found in these images, as indicated by the measured d‐spacing values of 3.31 and 5.24 Å, corresponding to (100) and (020) planes, respectively. Size distribution (Figure S5, Supporting Information) of our synthesized PNRs were analyzed and compared with previous works in Figure 3f, in which the PNRs produced using our method are obviously smaller in dimensions with thin widths (10.3 ± 3.8 nm) and short lengths (250 ± 156 nm), as obtained from the TEM analysis (Figure S5a, Supporting Information). With such small dimensions, the produced nanoribbons are able to possess more active sites at their edges and enhanced quantum confinement effects.^[^ ^2^ ^]^ This behavior has been observed in graphene nanoribbons (GNRs) as they display a finite bandgap when the widths below 10 nm.^[^ ^32^ ^]^ Notably, our produced PNRs display the narrowest widths in comparison with other methods, as seen in Figure 3f. Moreover, the shown data in this figure display a clear trend, suggesting that the aspect ratio for PNRs remains approximately constant in a wide range of lengths (10^1^–10^5^ nm). The vibrational modes and Raman peaks of as‐synthesized PNRs in this work are blue‐shifted (Figure 3h), with the suppressed *B*_2g_ peak, which is in good agreement with the observation by MacDonald et al.^[^ ^4^ ^]^ Pristine BP had peak positions for $A_{g,}^{1}\;B_{2g},\;\mathrm{and}\;A_{g}^{2}$ phonons, respectively at 361.3, 438.2 and 466.1 cm^−1^ while the PNRs only showed the peak positions for $A_{g,}^{1}\;\mathrm{and}\;A_{g}^{2}$ phonons at ≈ 363.0–363 cm^−1^ and ≈ 467.2–467.5 cm^−1^, respectively. The blue shift of $A_{g}^{1}/A_{g}^{2}$ and the suppressed *B*_2g_ peak observed in our synthesized PNRs was due to the decrease in thickness and dimensionality.^[^ ^27^, ^33^ ^]^

**Figure 4:**
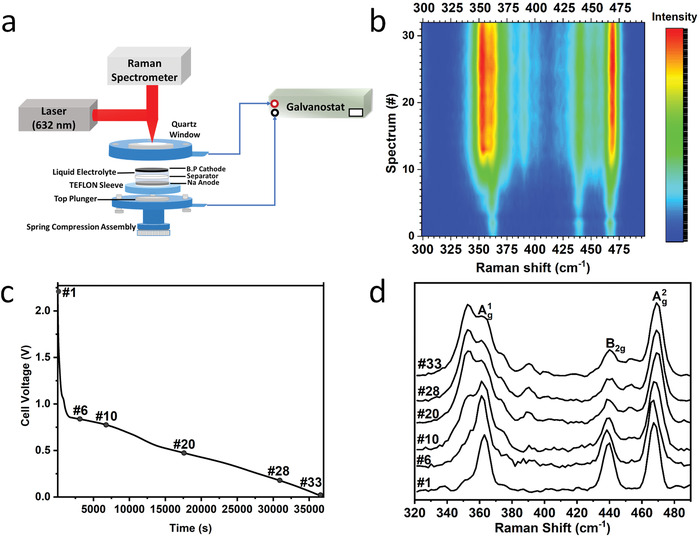
In situ Raman study of the PNR formation. a) In situ Raman electrochemical sodiation set‐up and graphic of mechanism PNRs production. b) Intensity color maps for in situ Raman spectra obtained at different times and degrees of sodiation using EC:PC electrolyte. c) Voltage profile during electrochemical Na intercalation process in liquid electrolyte. d) Comparison between the six Raman spectra taken during the in situ electrochemical experiment indicating the structural changes BP undergoes at the evolution of time. The six points labeled on discharge curve in (c) coincide with the time and voltages the spectra shown were taken.

To gain a better understanding of the mechanism of the electrochemical induced PNRs formation in BP, in situ Raman spectroscopy was performed (**Figure** **4**a) as it closely tracks the real‐time structural change of BP flake during the intercalation of Na^+^ in the ethylene carbonate:propylene carbonate (EC:PC) electrolyte environment. Figure 4b represents the spectra stack of BP taken at different intercalation levels using a dedicated electrochemistry in situ Raman cell set. We observed the downshifting of the active Raman phonons of BP: $A_{g}^{1}$ (out‐of‐plane armchair direction, ≈ 360 cm^−1^), *B*_2g_ (in‐plane zigzag direction, ≈ 437 cm^−1^) and $A_{g}^{2}$ (in‐plane armchair direction, ≈ 464 cm^−1^) up to the intercalation time of ≈ 15000 s (voltage > 0.55 V) as $A_{g}^{2}$ and *B*_2g_ phonons moved faster than $A_{g}^{1}$. This observation corresponds to the Na intercalation behavior that the structure of BP is preserved while the interlayer gap is disordered.^[^ ^31^, ^34^ ^]^ Further sodiation beyond ≈ 15000 s caused $A_{g}^{1}$ and *B*_2g_ phonon modes to exhibit satellite peaks at around ≈ 372 and 445 cm^−1^, respectively (Figure 4b), which may be attributed to the structural disorder along the zigzag direction in the area between nanoribbons. The in situ Raman spectra of six discharge times on the discharge curve (Figure 4c) are shown in Figure 4d, which clearly display the structural evolution of BP from selected spectra (#1, #6, #10, #20, #28, and #33). Spectra #1 (i.e., before the Na intercalation) and #33 (i.e., after the Na intercalation) were deconvoluted using the Lorentzian function, as shown in Figure S6, Supporting Information. We observed that spectrum #33 exhibited the signature modes of BP ($A_{g}^{1},\;A_{g}^{2}$, and weak *B*_2g_) and the remaining peaks closely resembled the peaks of red phosphorous (RP), as indicated by the comparison with the spectrum of RP (Figure S6c, Supporting Information). This strengthens the possibility of the partial disordering of BP structure due to lattice distortion as Na intercalation proceeded. Mitrovic et al.^[^ ^35^ ^]^ have reported the partial disordering of BP intercalation compounds (BPICs) into RP by diazonium salts. They found that the formation of amorphous phase in the reaction was facilitated by high concentration of intercalants (Na or K). Furthermore, the Raman spectra of the final product clearly exhibited peaks signature to BP and RP. Abellan et al.^[^ ^36^ ^]^ also observed Raman peak splitting, formation of new peaks and ribbons as they synthesized BPICs with Na and other alkali metals. They linked these outcomes to breaking of P—P bonds as intercalant atoms increased in BP sheets. Interestingly, additional Raman peaks observed by Watts et al.^[^ ^18^ ^]^ were attributed to symmetry distortions at the edges of as‐synthesized PNRs. Reordering of atoms around the edges of the nanoribbons and changes in the movement of atoms linked to the signature Raman modes cause the appearance of additional Raman tensor elements.^[^ ^37^ ^]^ The combined TEM and Raman results seem to suggest that there is a threshold of Na concentration and built‐in strain up to which the Na‐intercalated BP has a homogeneous, single‐phase structure (Figure S7a, Supporting Information). At higher concentration of Na^+^ ions above the threshold, a strain relaxation takes place, which leads to the phase segregation and alternating regions of unstrained BP (PNRs) and disordered phosphorous regions reach in Na (Figure S7b, Supporting Information). However, more detailed studies will be needed to understand this process.

However, it needs to be noted that previous systematic studies on the intercalation of BP with Li have shown different outcomes. The mechanism of intercalation of BP with Li^+^ ions as observed through Raman measurements is significantly different as *B*_2g_ and $A_{g}^{2}$ phonon modes steadily downshift 1.6 times faster than $A_{g}^{1}$ with the intensity of successive spectrum decreasing.^[^ ^29^ ^]^ The discrepancy in the behavior of the Raman spectra of BP under Na^+^ and Li^+^ intercalation influences is due the restriction of Na^+^ diffusion along the [100] armchair direction, which gives rise to columnar intercalation along the [001] zigzag direction.^[^ ^30^ ^]^

**Figure 5:**
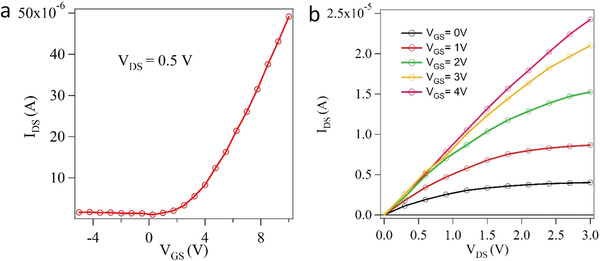
a) *I*_DS_–*V*_GS_ curves for an unseparated bundle of PNR at *V*_DS_ = 0.5 V; b) *I*_DS_–*V*_DS_ curves for varying *V*_GS_ values from 0 to 4 V in 1 V steps.

In **Figure** **5**, we show the electrical properties of a back‐gated FET based on an unseparated bundle of PNRs placed between aluminum source/drain with electrostatic electron doping by a back gate. As shown in Figure 5a, we supplied a fixed bias of 0.5 V between the source and the drain and swept the gate voltage from −5 to 10 V. The results demonstrate that the device state switched from “off” to “on” state with a current increase up to several orders for positive gate source bias (*V*_GS_) values. The measured transfer characteristics showing an increase of the current values when the gate voltage became increasingly positive is characteristic of the typical behavior of an n‐type FET due to the accumulation of the majority electron in an n‐type material at positive gate voltages. Figure 5b shows drain source current (*I*_DS_) versus drain source voltage (*V*_DS_) curves for varying *V*_GS_ values from 0 to 4 V in 1 V steps. As expected, *I*_DS_ increases with *V*_GS_ up to a certain point and becoming almost constant (plateau) as *V*_GS_ increases beyond that point. Normally, BP and black arsenic–phosphorous (b‐As*_x_* P_1−_ *_x_*) alloys are typical p‐type materials,^[^ ^38^ ^]^ and FET devices made out of thin layers of phosphorene,^[^ ^5^ ^]^ as well as thick flakes of BP^[^ ^39^ ^]^ show typical p‐type characteristics. Therefore, the n‐type behavior measured for our device is a direct evidence of the effective charge transfer and n‐type doping due to the Na intercalation into the structure.

## 3 Conclusions

Here, we reported a simple and feasible two‐step electrochemical intercalation method to produce PNRs with narrow widths of good uniformity (10.3 ± 3.8 nm). The prepared narrow PNRs show zigzag direction as well as the suppressed *B*_2g_ Raman mode. Interestingly, the FET device structure prepared from a bundle of PNRs showed the n‐type transistor behavior due to the effective charge transfer and n‐type doping induced by sodium intercalation. This work provides insights into a new synthesis approach of PNRs with confined width, paving the way toward the development of phosphorene and other highly anisotropic nanoribbon materials for high‐quality applications.

## 4 Experimental Section

Bulk BP was produced by means of chemical vapour transport (CVT) growth method from red phosphorous (500 mg, Sigma, > 97%) while Sn (20 mg, Alfa Aesar, 99.8%) and SnI_4_ (20 mg, Alfa Aesar, 95%) served as mineralization agents. The precursors were carefully measured into a quartz ampoule that was sealed at a vacuum of 10^−6^ Torr. At a temperature gradient of 50 °C, the sealed ampoule was annealed at 615 °C in a two‐zone furnace and precursors were placed at the hot end. Exhaustive steps for this process had been provided.^[^ ^29^, ^40^ ^]^

Electrochemical Na intercalation of BP was achieved in coin cells that were prepared with BP and Na metal as a cathode and an anode, respectively. Cathode pastes were prepared with the weight of the BP and carbon binder in the ratio 1:2, then the pastes were put on a stainless steel mesh (diameter 18 mm) and dried under vacuum at 150 °C for 3 h. The Na intercalation process was performed in liquid electrolyte medium that contained a mixture of 1 m of NaPF_6_ with a binary solution of EC:PC. The cut‐off voltage was set to 0.2 V under a current density of 30 µA cm^−2^. After the electrochemical intercalation step, the coin cell was opened in an inert atmosphere (glovebox) and the cathode was cleaned using DMF.

A dedicated electrochemical split cell with a quartz window manufactured by MTI Corporation was used for in‐situ electrochemical Na intercalion of BP. (Figure 4a). For the cathode, a similar weight ratio of BP to carbon binder used for the coin cells was adapted, and then ground to homogeneity. The paste was then placed on a stainless‐steel mesh to further enhance charge transfer and dried for 3 h at 150 °C. NaPF_6_ in EC:PC was used as electrolyte. The cell assembly was carefully carried out in an argon‐filled glovebox with both moisture and oxygen level less than 5 ppm.

After cycling was complete, the coin cells were taken apart in an argon‐filled glovebox and some of the cathode pastes were dispersed in a vial containing 2 mL of DMF. The vial was tightly closed and further made airtight with layers of parafilm. The vial was then sonicated for 10 min to clean off binder and separate bundles of PNRs into individual nanoribbons.

AFM imaging was performed using an Asylum Research MFP‐3D in AC‐mode. Images were ElectriTap300‐G, a tapping mode AFM probe with an overall platinum coating (Force constant 40 N m^−1^, resonant frequency 300 kHz, and a tip thickness of 4 µm) (Budgetsensors). All images were collected and processed (flattened and plane‐fit) using IgorPro software. Sample preparation for the AFM measurements was performed in a dry nitrogen glovebox. A drop of a suspension of PNRs in DMF was drop cast onto an SiO_2_ surface located on ≈ 5 mm square Si substrate. The drop was allowed to evaporate in the glovebox, leaving the residual solids from the suspension bound to the SiO_2_ surface. The residual solids form into random arrangements of blotches and streaks of various thicknesses.

An unseparated bundle of PNRs was used to fabricate an FET device structure, as the one shown schematically in Figure S8, Supporting Information. The aluminum contacts of the interdigitated contact pattern served as the source and the drain of the FET device while the p‐doped Si serves as the back gate. Electrical measurements were carried out using a Keithley 6487 picoammeter/voltage source which has the current resolution of 10 fA and a Keithley 2400 source‐measure unit to supply the gate voltage.

### Statistical Analysis

Using TEM images and AFM height profiles, 71 and 34 different nanoribbons, respectively, were analyzed. The distribution of width, length, and thickness on the nanoribbons was obtained. This data was also used to calculate the average values and standard deviations. These values obtained from the AFM measurements are consistent with the results obtained through the TEM analysis.

## Conflict of Interest

The authors declare no conflict of interest.

## Supporting information

Supporting Information

## Data Availability

The data that support the findings of this study are available from the corresponding author upon reasonable request.
