# Supplementary material for: Ultra‐Narrow Phosphorene Nanoribbons Produced by Facile Electrochemical Process
Source: Adv Sci (Weinh). 2022 Sep 6;9(31):2203148. doi: 10.1002/advs.202203148 (PMC9631066; doi:10.1002/advs.202203148)
Supplement: Supplementary file 1 — Supporting Information [file ADVS-9-2203148-s001.pdf]

Supporting Information

Ultra-Narrow Phosphorene Nanoribbons Produced by Facile Electrochemical Process

Usman O. Abu[#], Sharmin Akter[#], Bimal Nepal, Kathryn A. Pitton, Beth S. Guiton, Douglas R. Strachan, Gamini Sumanasekera, Hui Wang, Jacek B. Jasinski**

Usman O. Abu, Dr. Jacek B. Jasinski
Conn Center for Renewable Energy Research, University of Louisville
Louisville, KY, 40292, United States

Sharmin Akter, Dr. Hui Wang
Department of Mechanical Engineering, University of Louisville
Louisville, KY, 40292, United States

Bimal Nepal, Prof. Gamini Sumanasekera
Department of Physics and Astronomy, University of Louisville
Louisville, KY, 40292, United States

Kathryn A. Pitton, Prof. Beth S. Guiton
Department of Chemistry, University of Kentucky
125 Chemistry-Physics Building
Lexington, KY, 40506-0055, United States

Prof. Douglas R. Strachan
Department of Physics and Astronomy, University of Kentucky
177 Chemistry-Physics Building
Lexington, KY, 40506-0055, United States

*Corresponding authors: jacek.jasinski@louisville.edu, hui.wang.1@louisville.edu,
[#]These authors contributed equally.

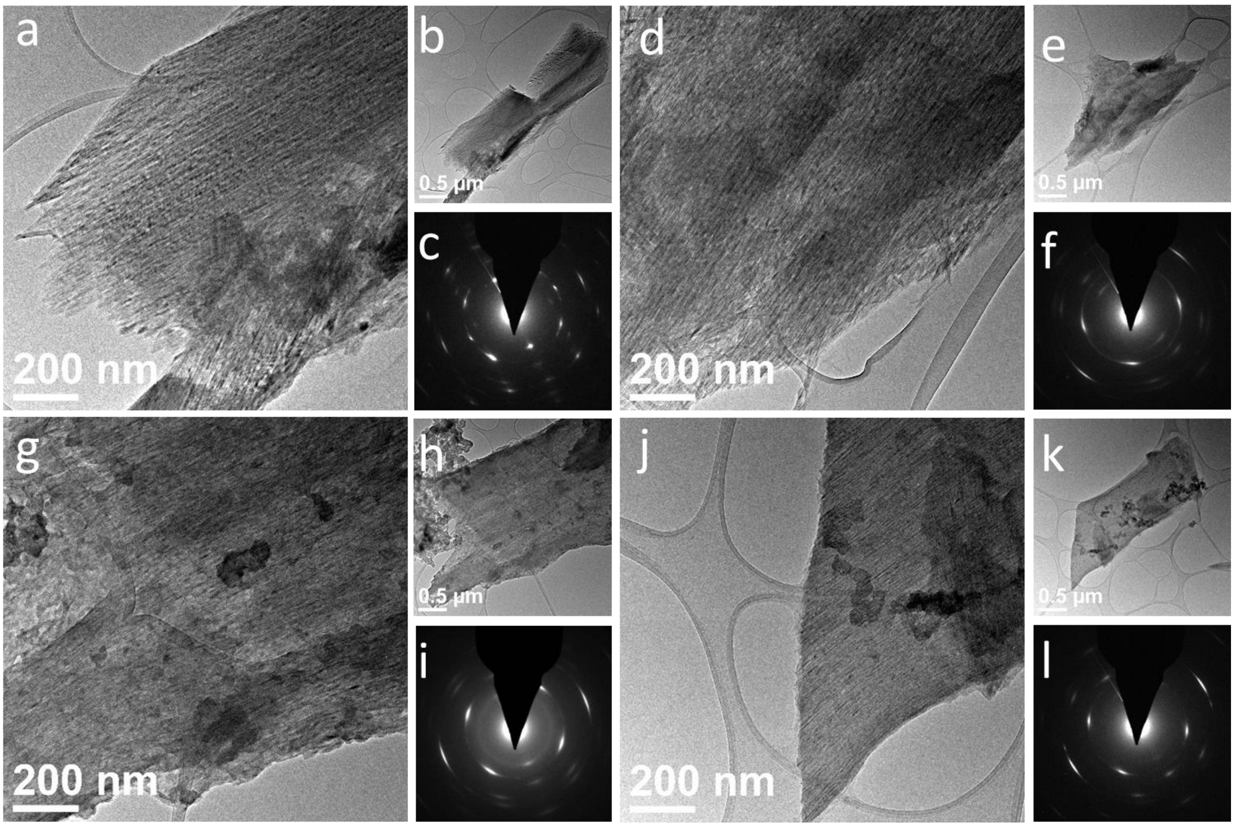

Figure S1. Sets of (a, d, g, j) high- and (b, e, h. k) low-magnification TEM images and the corresponding SAED pattern of 4 Na-intercalated BP flakes (c, f, i, l).

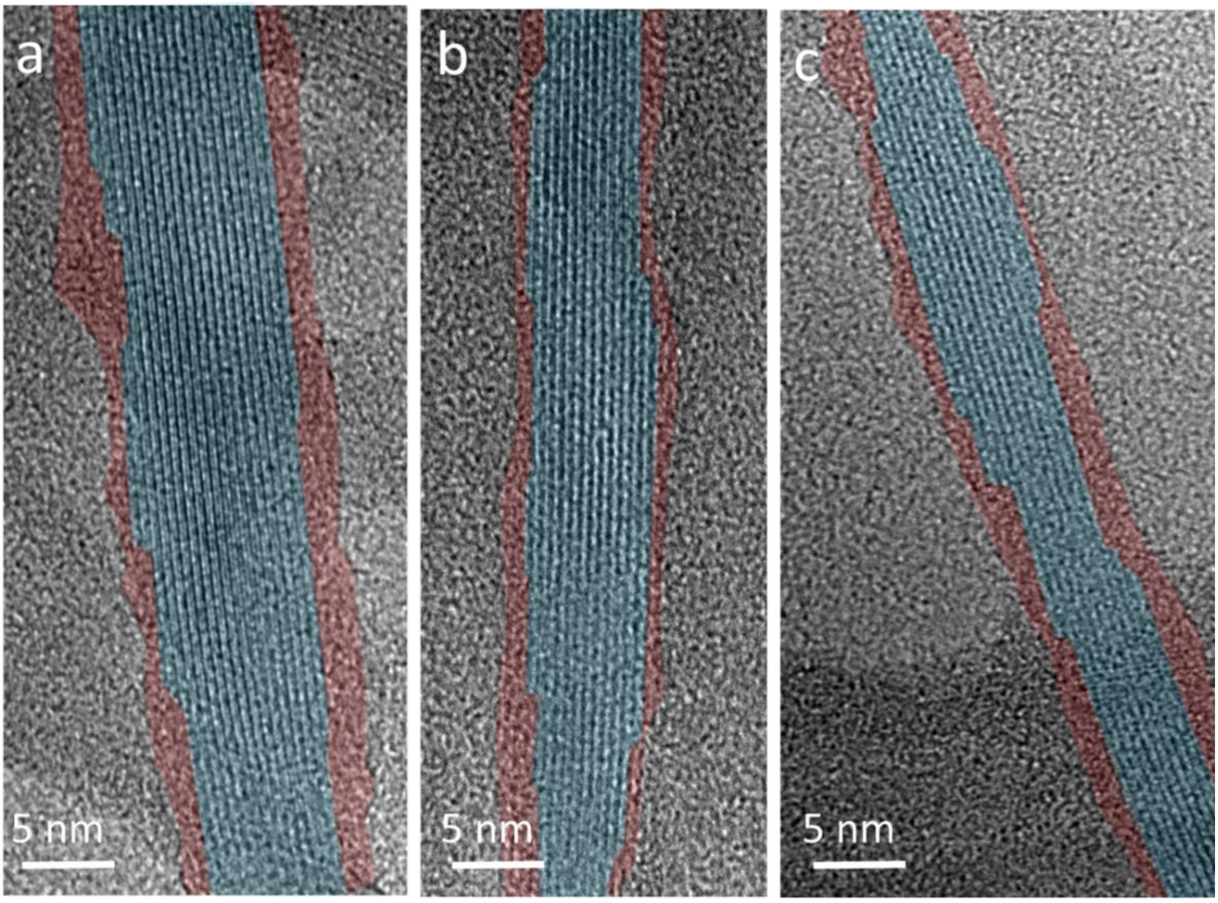

Figure S2. (a)-(c) HRTEM images of example PNRs. Pseudo-colors are used to indicate crystalline nanoribbons (blue) and amorphous edges (red).

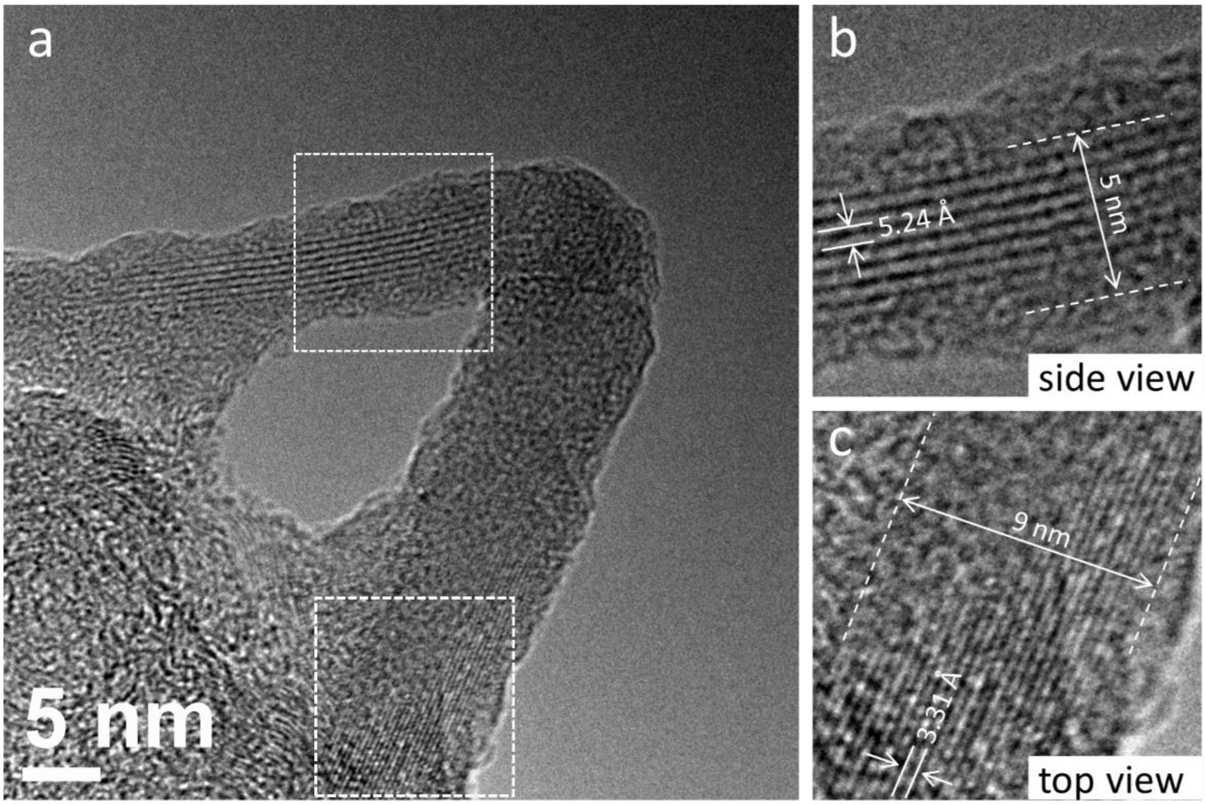

Figure S3. (a) HRTEM image of a folded nanoribbon showing the top and side views (b) the side & (c) top view of the nanoribbon from the areas marked with dashed boxes in (a).

AFM Analysis: To perform our AFM analysis, we focused on a relatively thin drop-cast streak shown in Figure S4a. Enlarging this region in Figure S4b shows that the streak is comprised of an accumulation of elongated features, which appear to be the phosphorene nanoribbons. The height profile in Figure S4c along the yellow line in Figure 4Sb shows that these elongated nanoribbons have a thickness of roughly $4\ nm$. Using such height profiles, we analyzed 34 different nanoribbons for this sample and obtained the distributions of thickness and length, as shown in Figure S4d and Figure S4e. Using these data, the thickness of the nanoribbons in this sample is computed to be $5.4 \pm 2.5\ nm$ while the length is computed to be $300 \pm 90\ nm$. These values for the thickness and length of the nanoribbons are consistent with the results obtained through the TEM analysis.

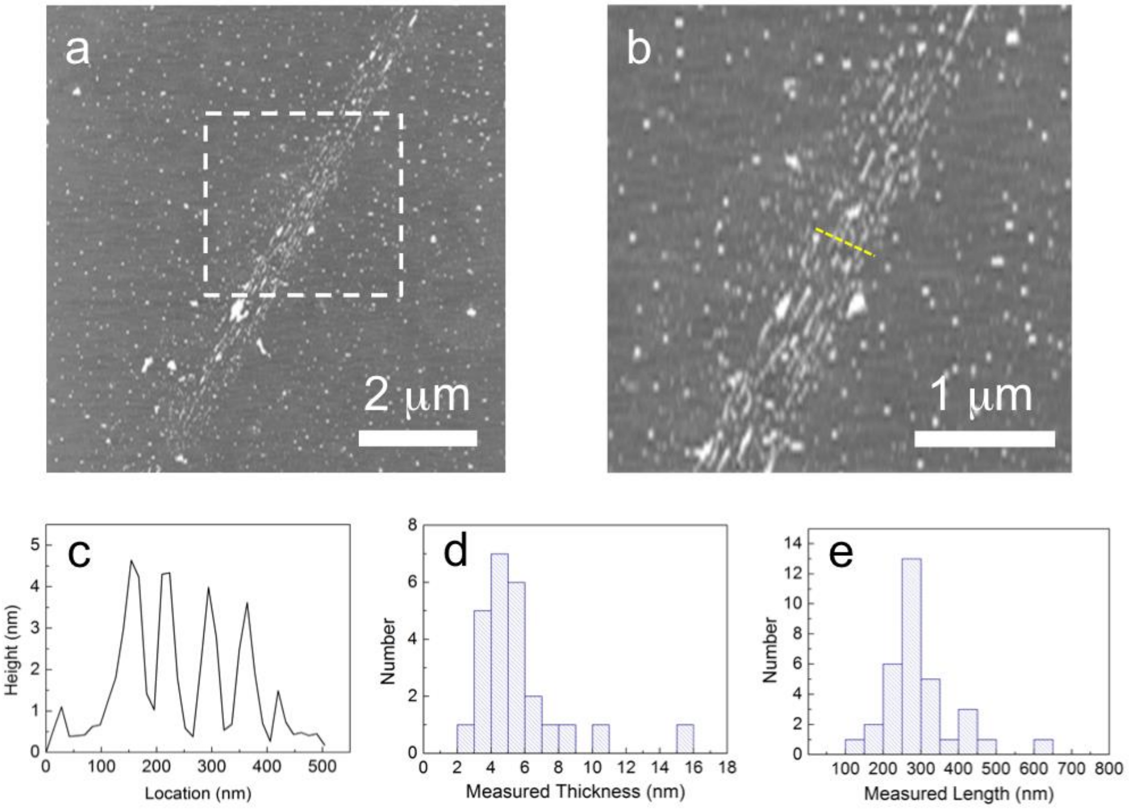

Figure S4. Atomic force microscopy (AFM) analysis of nanoribbons. (a) AFM image of a streak of nanoribbons formed through drop-casting that is directed from lower-left to upper right. (b) Enlarged image within dashed box in panel (a) showing that the streak consists of many elongated entities consistent with nanoribbons. (c) Height-scan profile taken along the dashed-yellow line in panel (b). (d-e) Thickness and length histogram analysis of 34 different elongated entities giving an average thickness of $5.4 \pm 2.5\ nm$ and length of $300 \pm 90\ nm$.

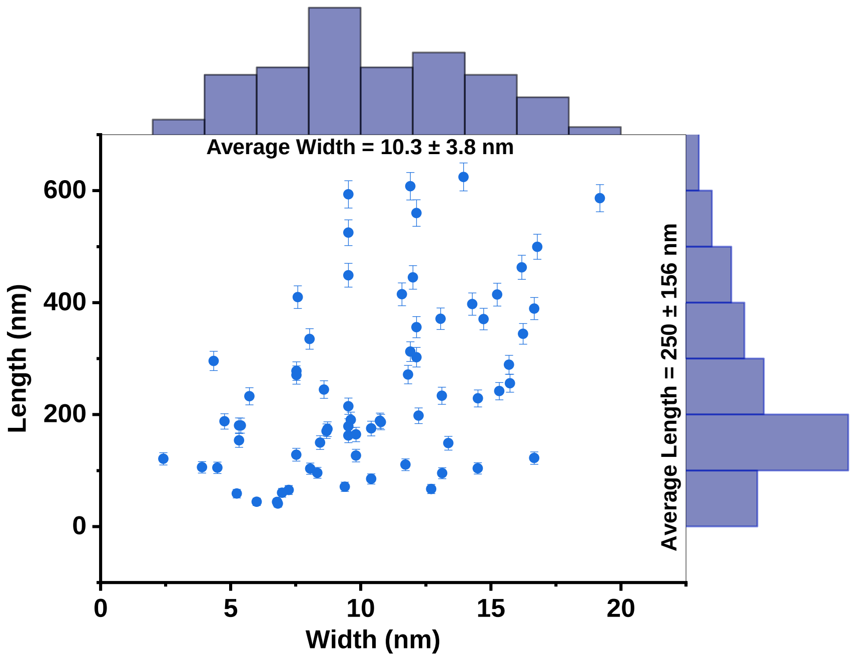

Figure S5-a. Statistical distribution of the lengths and widths of the as-synthesized PNRs obtained from the TEM analysis.

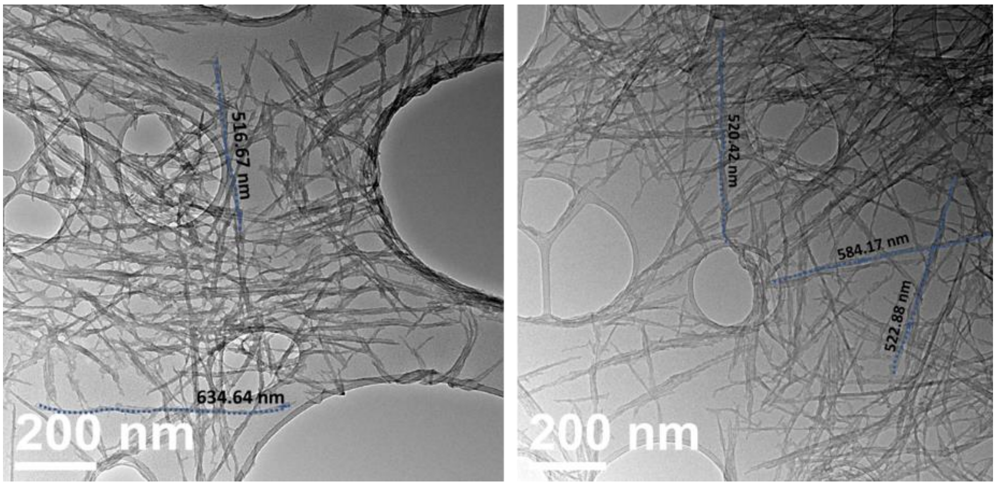

Figure S5-b. TEM images of PNRs with several nanoribbons longer than 500 nm indicated with blue lines.

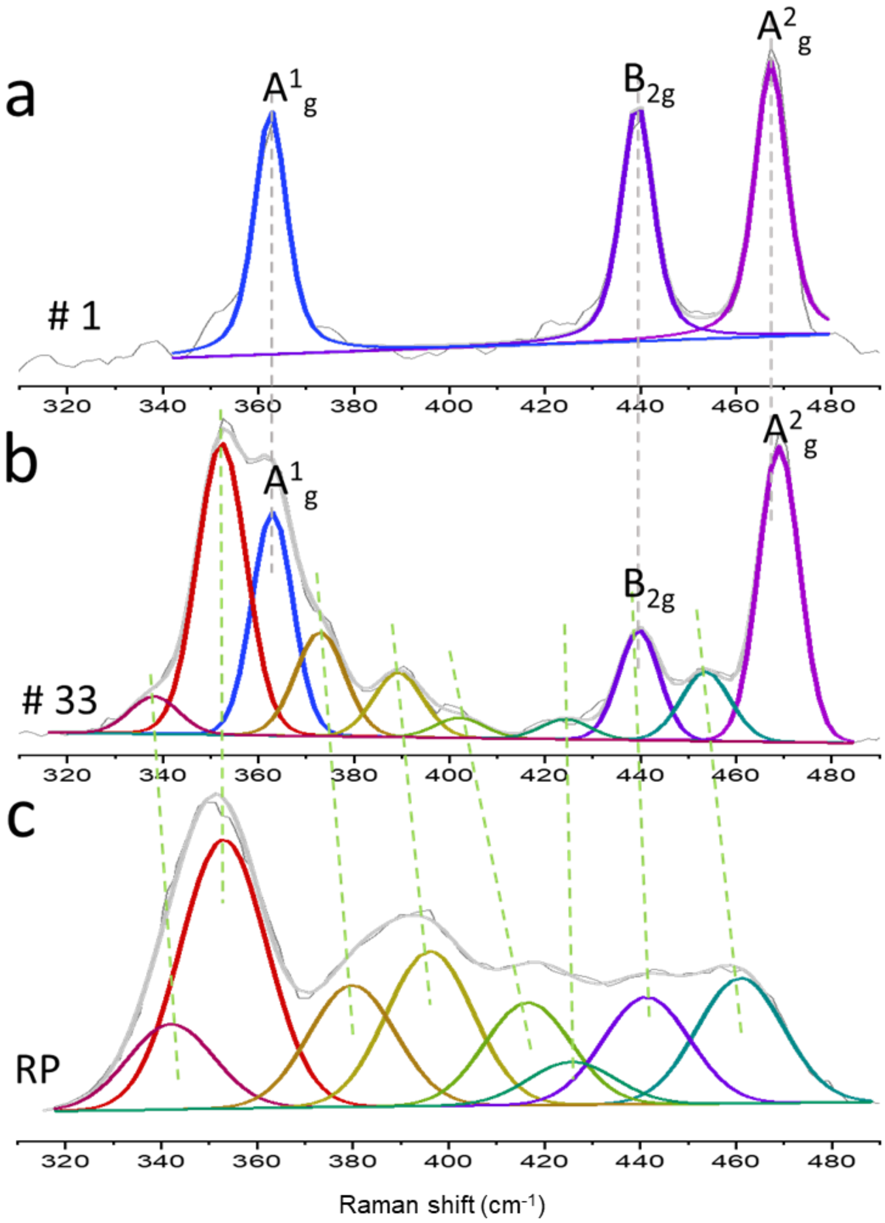

Figure S6. Raman spectra deconvolution. (a) Raman spectrum of the BP flake before intercalation. (b) Raman spectrum after the intercalation. In addition to BP peaks (originating from PNRs), there are several additional peaks coming from discorded RP-like regions between PNRs. Notice that the B_{2g} mode decreased significantly in agreement with the Raman data from exfoliated PNRs (see Fig. 3h). Note, that the B_{2g} in this sample can be overestimated because RP has a coincidental peak in the similar location (see Fig. S1(c)) (c) Raman spectrum of RP (data from Ref.[1]).

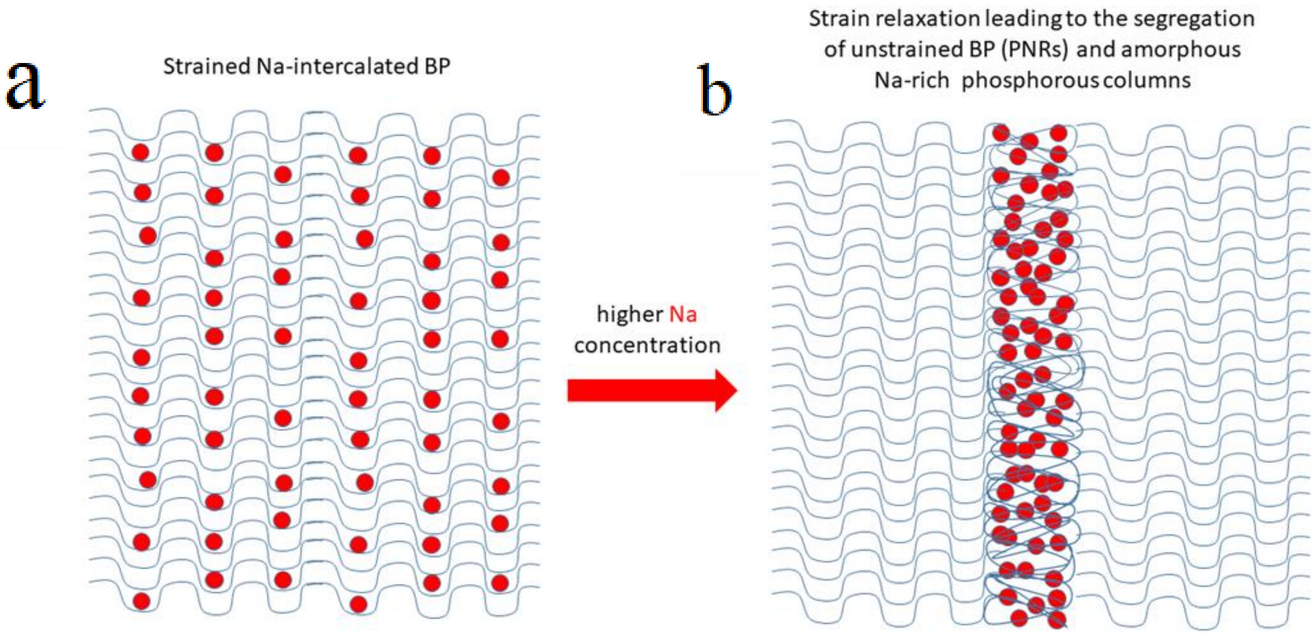

Figure S7. A schematic illustrating PNR formation mechanism. The possible mechanism involves tha the strain relaxiation between at a higher concentration of Na ions.

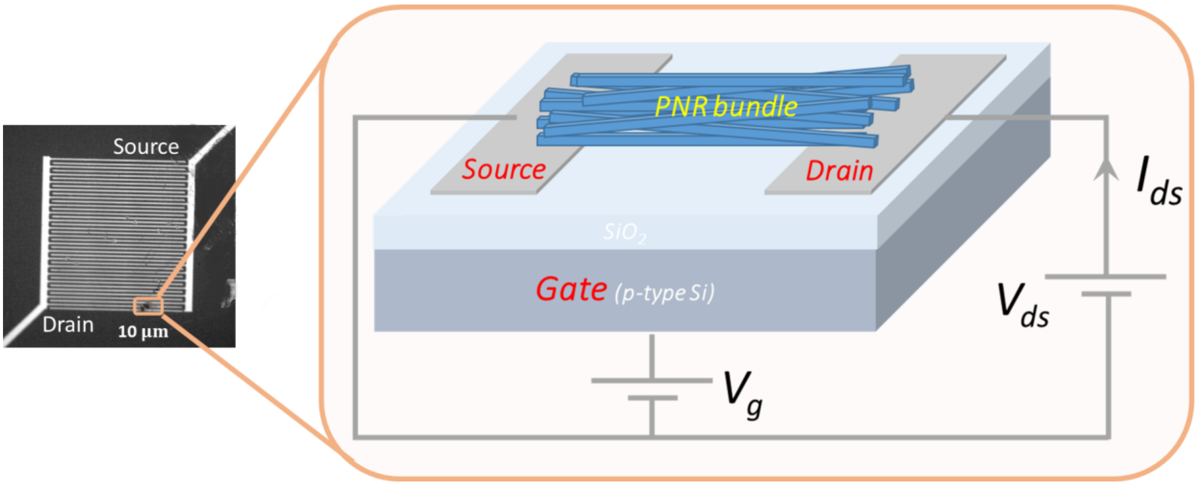

Figure S8. An optical micrograph and a schematic of a field effect transistor (FET) device structure, based on an unseperated bundle of PNRs.

References

[1] Q. Sun, X. Zhao, Y. Feng, Y. Wu, Z. Zhang, X. Zhang, X. Wang, S. Feng, X. Liu, *Inorganic Chemistry Frontiers* **2018**, 5, 669.
